# Separation and Concentration of Nitrogen and Phosphorus in a Bipolar Membrane Electrodialysis System

**DOI:** 10.3390/membranes12111116

**Published:** 2022-11-08

**Authors:** Xiaoyun Wu, Wanling Cai, Yuying Fu, Yaoxing Liu, Xin Ye, Qingrong Qian, Bart Van der Bruggen

**Affiliations:** 1 School of Safety and Environment, Fujian Chuanzheng Communications College, Fuzhou 350007, China; 2 College of Environmental and Resource Sciences, College of Carbon Neutral Modern Industry, Fujian Key Laboratory of Pollution Control & Resource Reuse, Fujian Normal University, Fuzhou 350007, China; 3 Key Laboratory of Urban Pollutant Conversion, Institute of Urban Environment, Chinese Academy of Sciences, Xiamen 361021, China; 4 Department of Chemical Engineering, ProcESS—Process Engineering for Sustainable System, KU Leuven, Celestijnenlaan 200F, B-3001 Leuven, Belgium; 5 Faculty of Engineering and the Built Environment, Tshwane University of Technology, Private Bag X680, Pretoria 0001, South Africa

**Keywords:** phosphorus, nitrogen, electrodialysis, bipolar membrane, concentration

## Abstract

Struvite crystallization is a successful technique for simultaneously recovering PO_4_^3−^ and NH_4_^+^ from wastewater. However, recovering PO_4_^3−^ and NH_4_^+^ from low-concentration solutions is challenging. In this study, PO_4_^3−^, NH_4_^+^, and NO_3_^−^ were separated and concentrated from wastewater using bipolar membrane electrodialysis, PO_4_^3−^ and NH_4_^+^ can then be recovered as struvite. The separation and concentration of PO_4_^3−^ and NH_4_^+^ are clearly impacted by current density, according to experimental findings. The extent of separation and migration rate increased with increasing current density. The chemical oxygen demand of the feedwater has no discernible impact on the separation and recovery of ions. The migration of PO_4_^3−^, NH_4_^+^, and NO_3_^−^ fits zero-order migration kinetics. The concentrated concentration of NH_4_^+^ and PO_4_^3−^ reached 805 mg/L and 339 mg/L, respectively, which demonstrates that BMED is capable of effectively concentrating and separating PO_4_^3−^ and NH_4_^+^. Therefore, BMED can be considered as a pretreatment method for recovering PO_4_^3−^ and NH_4_^+^ in the form of struvite from wastewater.

## 1. Introduction

Phosphorous and nitrogen are extensively used in industrial and agricultural operations as a result of the growth of the global economy, and their subsequent release into the environment has led to environmental issues such as eutrophication and red tides [1]. Phosphorous and nitrogen are vital constituents of fertilizers in agricultural production [2]. The primary sources of phosphorus are phosphate rocks [3], from which more than 90% of phosphorus used in commercial fertilizers and animal feed is obtained [4]. However, high-quality phosphate rocks have become increasingly scarce and expensive, yet they are abundant in some nations [5]. By the end of the 21st century, the known worldwide phosphate rock reserves are anticipated to run out [6]. This has led to widespread concerns about the health of the ecosystem, future phosphorus supplies, and food production. As a result, some nations have recently started making efforts to recover phosphorus from wastewater rather than its removal [7], especially in nations with limited to no phosphate rock resources [8]. In contrast to phosphorus, nitrogen resources are plentiful and have not been shown to be in short supply globally. They can also form a closed-circuit cycle in the natural environment. However, the usage of nitrogen fertilizer and its subsequent loss in agriculture has created a significant imbalance in the global nitrogen cycle. Each year, some 100 million tons of synthetic ammonia are produced from chemical fertilizers, and the majority of this ammonia is not taken up by plants, leaving many areas with surplus nitrogen. Therefore, it is essential to minimize phosphorus and nitrogen discharge and optimize their recovery from wastewater in accordance to the environmental protection and “circular economy” strategies being followed.

An effective technique for concentrating anions and cations from salty wastewater is electrodialysis [9]. A bipolar membrane (BPM) allows to split water into H^+^ and OH^−^ in the presence of an electrical field [10]. Bipolar membrane electrodialysis (BMED), which combines electrodialysis with a bipolar membrane to treat saline wastewater and convert anions and cations to the equivalent acid and base, has attracted increasing attention in recent years [11]. In prior work [12,13], BMED was employed to successfully recover HCl and NH_3_∙H_2_O from simulated NH_4_Cl. BMED has not been for explored PO_4_^3−^ recovery, while nanofiltration and forward osmosis are frequently used to separate and concentrate PO_4_^3−^ from wastewater [14]. In order to separate the enriched anions into monovalent and multivalent ions, as well as to increase the purity of the recovered anions, a combined system of the monovalent selective anion exchange membranes (MVA) and selectrodialysis (SED) was developed. A selectrodialysis system was utilized to concentrate PO_4_^3−^ in water and achieve the separation of PO_4_^3−^ and Cl^−^, significantly enhancing the purity of recovered PO_4_^3−^ [15].

This work explores a selective BMED (SBMED) that integrates BMED and MVA to separate and concentrate PO_4_^3−^, NH_4_^+^, and NO_3_^−^ from wastewater, while also separating PO_4_^3−^ and NO_3_^−^. This system can obtain high-purity PO_4_^3−^, which may be utilized as a pretreatment system for recovering NH_4_^+^ and PO_4_^3−^ in the form of struvite, in addition to recovering NH_4_^+^ from wastewater in the form of NH_3_·H_2_O. How do variables like current density and organic matter content impact the effectiveness of NH_4_^+^ and PO_4_^3−^ separation and concentration? What are the factors that affect its migration rate? How effectively does MVA separate NO_3_^−^ and PO_4_^3−^? What are the kinetics of NH_4_^+^ and PO_4_^3−^ removal? These questions remain unanswered.

Due to the fact that the electrolyte concentration has a substantial impact on energy consumption, the influence of the electrolyte concentration on cell voltage was initially examined in this study. Second, the impact of current density on the separation rate, concentration, and migration rate of PO_4_^3−^, NH_4_^+^, and NO_3_^−^ was examined. Third, the kinetics of PO_4_^3−^, NH_4_^+^, and NO_3_^−^ migration were studied. Fourth, the relationship between the concentration of organic matter and the rates of separation and concentration of PO_4_^3−^, NH_4_^+^, and NO_3_^−^ was examined. Finally, the concentration capacity of the SBMED for PO_4_^3−^ and NH_4_^+^ was examined.

## 2. Materials and Methods

### 2.1. Materials and Agents

All the analytical grade chemical reagents utilized in this study were sourced from Sinopharm Chemical Reagent Co. (Shanghai, China). Table S1 provides a complete listing of the membranes employed in this study(see Supplementary Materials). Pure water produced by a water-purification system (Purelab^®^ Pulse 2, Elga, High Wycombe, England) was used throughout the experimentation. The solution containing PO_4_^3−^ (200 mg/L), NH_4_^+^ (100 mg/L), and NO_3_^−^ (20 mg/L) was prepared by dissolving NH_4_Cl, Na_3_PO_4_, and NaNO_3_ in pure water.

### 2.2. Experimental Setup

**Figure 1:**
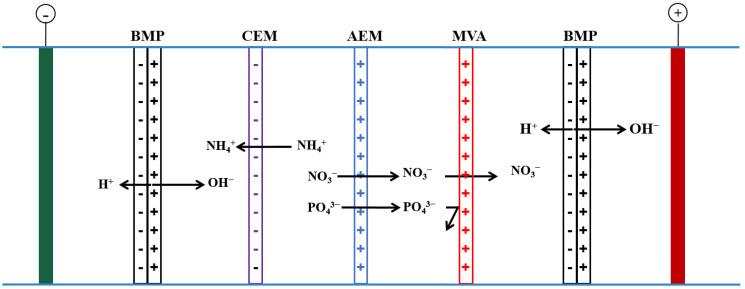
Schematic diagram of experimental equipment. CC: cathode compartment; AWC: ammonia compartment; WWC: wastewater compartment; PC: phosphate compartment; NAC: nitric acid compartment; AC: anode compartment.

The experimental setup is depicted in Figure 1. Two titanium plates were employed as the anode and cathode, respectively, and the BPM, cation exchange membrane (CEM), anion exchange membrane (AEM), and MVA were all placed into the BMED. From left to right, the device consists of a cathode compartment (CC), an ammonia water compartment (AWC), a wastewater compartment (WWC), a phosphate compartment (PC), a nitric acid compartment (NAC), and an anode compartment (AC). Each compartment had an effective volume of 45 mL, and the membrane’s effective area was 12 cm^2^. A sodium sulfate (Na_2_SO_4_) solution with a 0.8 mol/L concentration was utilized as the electrolyte during the experiment. Every 10 min, the voltage value was recorded. A current density of 0.5, 1.0, 2.0, 3.0, and 4.0 mA/cm^2^ was used during the experiments according to a previous study [16]. At pre-determined intervals of 10, 20, 30, 40, 60, 80, 100, and 120 min, water samples were taken.

Under the influence of the electric field, during the experiment, NH_4_^+^ in the WWC migrates into the AWC to form NH_3_·H_2_O with the OH^−^ generated by the bipolar membrane, and PO_4_^3−^ and NO_3_^−^ migrate to PC via the AEM, NO_3_^−^ then migrates to the NAC to form HNO_3_ with H^+^ generated by the bipolar membrane. MVA achieves the separation and concentration of NO_3_^−^ and PO_4_^3−^ by retaining PO_4_^3−^ in the PC.

### 2.3. Calculation Methods

According to the Chinese standard procedure, a UV-vis spectrophotometer was used to measure PO_4_^3−^, NH_4_^+^, and NO_3_^−^ [17]. The separation and concentration rates of PO_4_^3-^, NH_4_^+^, and NO_3_^−^ were determined through Equations (1) and (2). Furthermore, Equations (3) and (4) were used to compute the energy consumption and current efficiency for the separation and enrichment of PO_4_^3−^, NH_4_^+^, and NO_3_^−^, respectively [18], and Equation (5) was used to calculate the ion migration rate.

(1) $$\mathrm{Separation}\;\mathrm{rate}\;(\%)=\frac{C_{o}-C_{t}}{C_{o}}\times 100$$

(2) $$\mathrm{Concentration}\;\mathrm{rate}\;(\%)=\frac{C_{r}}{C_{o}}\times 100$$

(3) $$\mathrm{Specific}\;\mathrm{energy}\;\mathrm{consumption}\;(\mathrm{SEC},\;\mathrm{kW}\cdot h/g)=\int \frac{UId_{t}}{(C_{o}-C_{t})V}\times 10^{-3}$$

(4) $$\mathrm{Current}\;\mathrm{efficiency}\;\eta \;(\%)=\frac{(C_{o}-C_{t})VFn}{60ItM}\times 100$$

(5) $$\mathrm{Ions}\;\mathrm{migration}\;\mathrm{rate}\;(g/h/m^{2})=\frac{60(C_{o}-C_{t})V}{St}$$

where *C_o_* is the initial concentration of PO_4_^3−^, NH_4_^+^, and NO_3_^−^ in the WWC at the initial time, g/L; *C_t_* is the concentration of PO_4_^3−^, NH_4_^+^, and NO_3_^−^ at time t, g/L; *C_r_* is the concentration of PO_4_^3−^, NH_4_^+^, and NO_3_^−^ in PC, AWC, and NAC at time t, g/L; *V* is wastewater volume, L; *t* is the experimental time, min; *U* is the BMED voltage, V; *I* is the current, A; *F* is Faraday’s constant, 96,500 C/mol; *M* is the molar mass of PO_4_^3−^, NH_4_^+^, and NO_3_^−^, g/mol; *n* is the ionic valence state of PO_4_^3−^, NH_4_^+^, and NO_3_^−^; *S* is the effective membrane area, m^2^.

### 2.4. Analysis of Reaction Kinetic Process

The reaction kinetics is mainly used to study a dynamic non-equilibrium system where the concentration of the reactants decreases with time while the concentration of the products increases. Equation (6) displays the related equation. The kinetic equation is frequently separated into zero-, first-, and second-order kinetics in accordance with the various reaction orders (Equations (7)–(9)) [19].

(6) $$-\frac{dC}{dt}=kC^{n}$$

(7) $$C_{t}-C_{o}=-kt$$

(8) $$\ln C_{t}-\ln C_{o}=-kt$$

(9) $$\frac{1}{C_{t}}-\frac{1}{C_{o}}=kt$$

where *C* is the reactant concentration, *C_t_* is the reactant concentration at time *t*, *C_o_* is the initial reactant concentration, *t* is the reaction time, *k* is the coefficient of kinetics, and *n* is the reaction order.

## 3. Results and Discussion

### 3.1. Influence of Electrolyte Concentration on Cell Voltage

The influence of the electrolyte concentration on cell voltage was examined with a current density of 2.0 mA/cm^2^ and electrolyte Na_2_SO_4_ concentrations of 0.2, 0.4, 0.6, 0.8, 1.0, 1.2, and 1.4 mol/L in each compartment.

**Figure 2:**
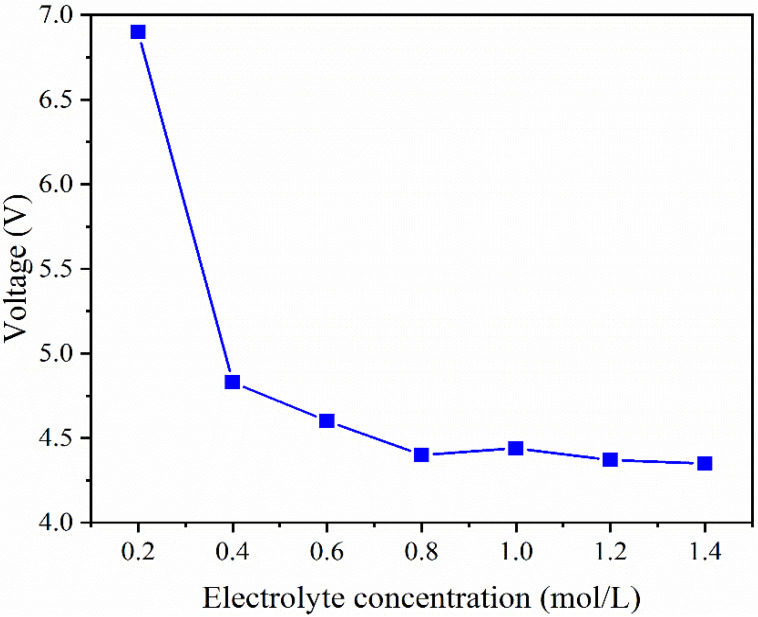
Cell voltage variation of cell voltage with Na_2_SO_4_ concentration.

It is evident from Figure 2, that when the concentration of electrolyte increased, the cell voltage first dropped rapidly before stabilizing. As the concentration of Na_2_SO_4_ was increased from 0.2 to 0.8 mol/L, the cell voltage dropped from 6.9 to 4.4 V. The cell voltage fell to 4.35 V as the concentration of Na_2_SO_4_ was further increased further from 0.8 to 1.4 mol/L. These are primarily driven by the fact that the system impedance and current are connected to the cell voltage. Since the current is constant (0.024 A), the system impedance was used to regulate the cell voltage. According to Figure 1, the system impedance is mostly made up of compartments and membranes. The cell voltage is mostly correlated with the compartment impedance during the experiment because the impedance of each membrane is also a consistent value. The conductivity of each compartment increased (reduced impedance) when the electrolyte content increased from 0.2 to 0.8 mol/L, thus, causing a decrease in impedance and cell voltage. Theoretically, once the electrolyte concentration was increased from 0.8 to 1.4 mol/L, the impedance of each compartment would decrease even more. However, the impedance decrease brought on by the increase of electrolyte concentration from 0.8 to 1.4 mol/L is negligible in comparison to the system’s overall impedance [20]. Consequently, the cell voltage did not change significantly as the electrolyte concentration increased from 0.8 to 1.4 mol/L. In the subsequent experiment, 0.8 mol/L of Na_2_SO_4_ was utilized as the electrolyte in all compartments aside from the WWC to minimize the amount of electrolyte as much as possible and achieve a lower cell voltage.

### 3.2. Effect of Current Density on Ions Separation and Concentration

#### 3.2.1. Effect of Current Density on the Separation of NH_4_^+^, PO_4_^3−^, and NO_3_^−^

**Figure 3:**
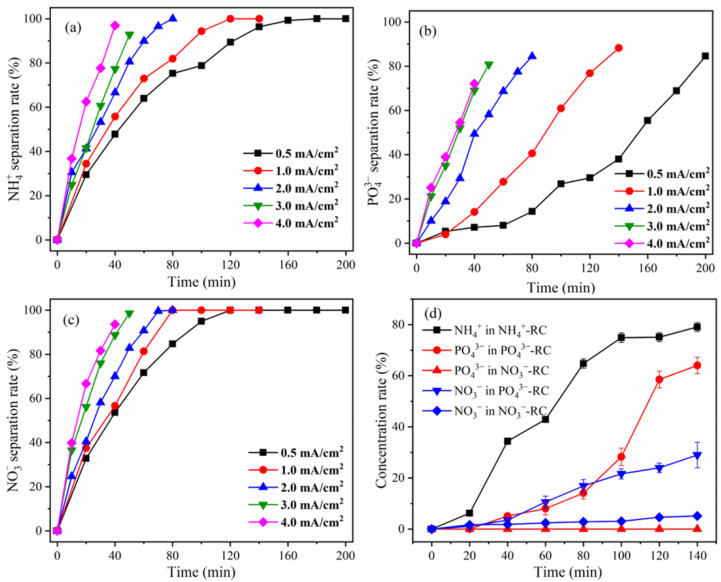
Effect of current density on the separation rate of (**a**) NH_4_^+^, (**b**) PO_4_^3−^, (**c**) NO_3_^−^, and (**d**) the concentration rate of NH_4_^+^, PO_4_^3−^, and NO_3_^−^ in different compartments under the condition of current density 1.0 mA/cm^2^.

According to the expectations, the separation rates of NH_4_^+^, PO_4_^3−^, and NO_3_^−^ increased over time (Figure 3a,b). Additionally, the current density increased the rate of separation of NH_4_^+^, PO_4_^3−^, and NO_3_^−^. The extents of NH_4_^+^ separation were 47.8% (0.5 mA/cm^2^), 55.8% (1.0 mA/cm^2^), 66.6% (2.0 mA/cm^2^), 77.3% (3.0 mA/cm^2^), and 97.0% (4.0 mA/cm^2^) after 40 min; that of PO_4_^3−^ were 7.2%, 14.1%, 49.4%, 69.0% and 72.1% and the extents of NO_3_^−^ separation were 53.7, 56.7, 70.0, 88.7, and 93.6%, respectively. This is due to the fact that when the current density is increased, the electric field force acting on ions is increased, causing ion migration to accelerate [18]. However, as time passes, a high separation rate can still be achieved with a modest current density. For instance, when the current density was 0.5 and 1.0 mA/cm^2^, the extents of NH_4_^+^ separation were 96.4 and 100% respectively, and PO_4_^3−^ separation rates were 38.0 and 88.3%, respectively, while the NO_3_^−^ separation rates were 100% after 140 min.

#### 3.2.2. Concentration Rate of NH_4_^+^, PO_4_^3−^, and NO_3_^−^

Figure 3d demonstrates the concentration rate of NH_4_^+^, PO_4_^3−^, and NO_3_^−^ in each compartment when the current density was 1.0 mA/cm^2^. At 140 min, the NH_4_^+^ concentration rate in AWC was 79.1%, which was lower than the NH_4_^+^ separation rate. This is primarily caused by the following two reasons. First, the CEM has a large number of anionic functional groups [21], which absorbed part of NH_4_^+^; second, when the pH is high, the equilibrium reaction of Equation (10) occurs in the AWC, and some NH_4_^+^ is eliminated as NH_3_ as a result. This could slow down the rate at which NH_4_^+^ builds up in the WWC. According to Figure 3d, the PC had a concentration rate of PO_4_^3−^ of 64.1%, whereas no PO_4_^3−^ was detected in the NAC. It is imperative to mention here that the concentration of PO_4_^3−^ is also significantly lower than the separation rate, which is primarily because some of PO_4_^3−^ was adsorbed by AEM [22]. Since no PO_4_^3−^ was detected in the NAC, MVA had good retention of PO_4_^3−^. In terms of NO_3_^−^ recovery, at 140 min, 29.0% NO_3_^−^ was recovered in the PC and 5.2% NO_3_^−^ was recovered in the NAC. The NO_3_^−^ concentration rate in the PC and the NAC was significantly lower than the NO_3_^−^ separation rate, this is primarily due to AEM and MVA adsorbing the majority of NO_3_^−^.

NH_4_^+^ + OH^−^ ⇌ NH_3_·H_2_O ⇌ NH_3_↑ + H_2_O (10)

#### 3.2.3. Effect of Current Density on Cell Voltage and Energy Consumption

**Figure 4:**
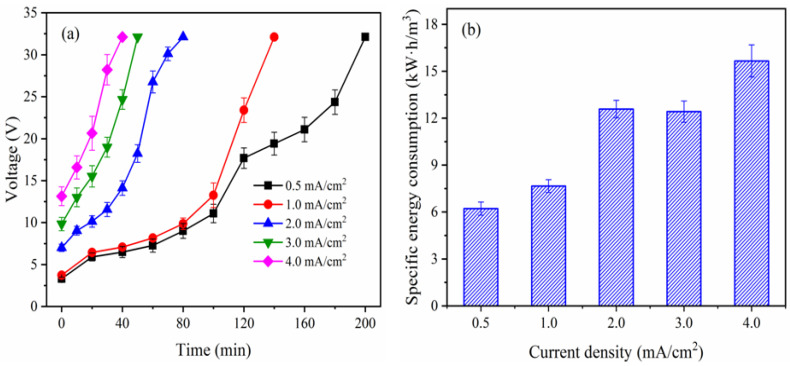
(**a**) Voltage and (**b**) energy consumption under different current density.

According to Figure 4a, the cell voltage increased rapidly with time, this is because the conductivity in the WWC had a decrease within the corresponding time frame (Figure S1) due to NH_4_^+^ migrating from the WWC to the AWC and PO_4_^3−^ and NO_3_^−^ migrating from the WWC to the PC, respectively, under the influence of the electric field [23]. Additionally, it was discovered that the cell voltage increased with current density. This is because the cell voltage directly correlates with both current and impedance, and as current increased, so did voltage. Likewise, as current density increased, ions moved faster from the WWC to the AWC and PC, increasing the cell voltage. In contrast, the conductivity in WWC decreased with an increase in current density (Figure S1) [24]. The specific energy consumption was calculated after 40 (4.0 mA/cm^2^), 50 (3.0 mA/cm^2^), 80 (2.0 mA/cm^2^), 140 (1.0 mA/cm^2^), and 200 min (0.5 mA/cm^2^) since a similar PO_4_^3−^ separation rate was observed during these times under varying current densities. The specific energy consumption increased with current density, as can be seen in Figure 4b. Equation (3) states that the specific energy consumption is inversely proportional to the quantity of eliminated ions and directly proportional to cell voltage, current, and time. The cell voltage and current increased with current density, which could in return increase the specific energy consumption. However, the increase in current density reduces the time needed for the ions separation rate to reach a given level, which helps to conserve specific energy. Meanwhile, a different quantity of PO_4_^3−^, NH_4_^+^ and NO_3_^−^ was removed under the condition of a varying current density. In light of the aforementioned variables, the specific energy consumption depicted in Figure 4b was derived.

#### 3.2.4. Effect of Current Density on the pH in AWC and NAC

**Figure 5:**
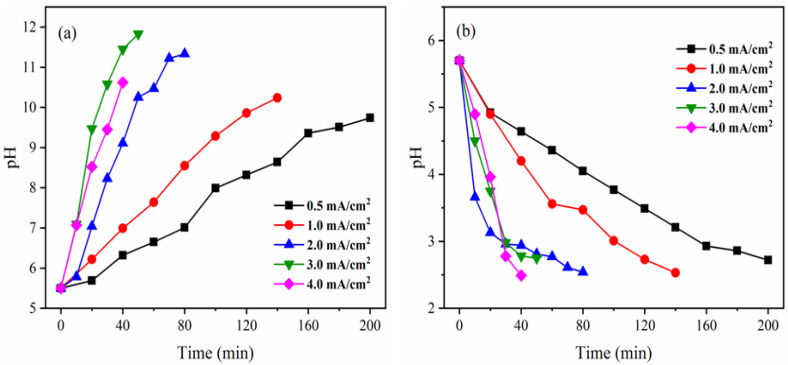
Effect of current density on (**a**) solution pH of ammonia compartment and (**b**) nitric acid compartment.

Figure 5 displays the pH variation in AWC and NAC. As time and current density increased, the pH in AWC increased while the pH in NAC decreased. The fundamental reason for this is that the electric field force caused the water in the bipolar membrane to dissociate into OH^-^ and H^+^ [25]. The OH^−^ migrated into the AWC and combined with NH_4_^+^ to form NH_3_·H_2_O and the H^+^ migrated into NAC and combined with NO_3_^−^ to form HNO_3_. The pH of AWC increased while the pH of NAC decreased as time passed because more OH^−^ and H^+^ were produced. Furthermore, the hydrolysis rate of the bipolar membrane increased with current density, subsequently, the pH in AWC increased and the pH in NAC decreased. At 40 min, the pH levels in AWC were 6.32 (0.5 mA/cm^2^), 6.99 (1.0 mA/cm^2^), 9.11 (2.0 mA/cm^2^), 11.45 (3.0 mA/cm^2^), and 10.62 (4.0 mA/cm^2^), those in NAC were 4.64, 4.20, 2.94, 2.78, and 2.49. From Figure 5a, it was also found that the pH in AWC dropped from 11.45 to 10.62 as the current density increased from 3.0 to 4.0 mA/cm^2^ after 40 min. Theoretically, when current density increased, OH^−^ generation increased as well, raising the pH in the AWC [14]. However, as Equation 10 demonstrates, there is a reaction equilibrium in the AWC. As the concentration of NH_4_^+^ in the AWC increases, more OH^−^ will react with it to generate NH_3_·H_2_O, which will lower the pH.NH_3_·H_2_O. According to Figure 3a, the separation rates of NH_4_^+^ after 40 min were 77.3% and 97.0% when the current densities were 3.0 and 4.0 mA/cm^2^, respectively. This emphasizes that compared with a current density of 3.0 mA/cm^2^, more NH_4_^+^ entered the AWC and combined with OH^−^ while under the condition of current density at 4.0 mA/cm^2^, a decrease of the pH in AWC was observed as a result of the increase in current density from 3.0 to 4.0 mA/cm^2^. Thus, it can be incurred that, NH_4_^+^ is primarily concentrated in the form of NH_3_·H_2_O, while NO_3_^−^ is concentrated in the form of HNO_3_, and PO_4_^3−^ is concentrated in the PC.

Taking into account energy consumption and ion separation rate, a current density of 1.0 mA/cm^2^ was used for the subsequent experiment. However, in a field application, if a short time is needed, a high current density might be used to reduce pretreatment time and boost ion removal.

### 3.3. Effect of Organic Matter Concentration

**Figure 6:**
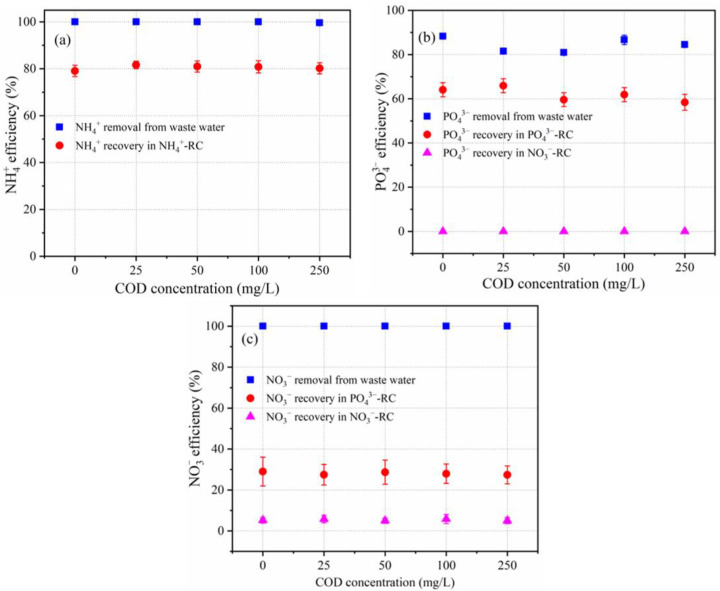
Effect of COD concentration on the separation and concentration of (**a**) NH_4_^+^, (**b**) PO_4_^3−^ and (**c**) NO_3_^−^.

Typically, organic matter is present in nitrogen- and phosphorus-containing wastewater. A series of simulated wastewater samples with various chemical oxygen demand (COD, which was prepared with glucose) concentrations (0, 25, 50, 100, and 250 mg/L) were used in order to evaluate the impact of organic matter on the separation and concentration of NH_4_^+^, PO_4_^3−^, and NO_3_^−^. Figure 6 depicts the separation and concentration of NH_4_^+^, PO_4_^3−^, and NO_3_^−^ in each compartment during 140 min. The separation and concentration rates of NH_4_^+^ were 100% and 80%, respectively, under varying COD concentrations.

The separation rate of PO_4_^3−^ in the WWC was 80–90%, subsequently, the concentration rate of PO_4_^3−^ in the PC was observed to be 60–65%, while no PO_4_^3−^ was detected in the NAC. The NO_3_^−^ separation rate in the WWC was 100%, and its concentration rate in the PC and the NAC was in the range of 27.4–29.0% and 5.0–5.8%, respectively. This suggests that almost the same separation and concentration of NH_4_^+^, PO_4_^3−^, and NO_3_^−^ were achieved under varying COD conditions, indicating that COD had essentially no effect. This may be because organic matter existed in a non-ionic state and would not migrate during the electrodialysis process, and had no migration competition for ion migration. The cause of the lower concentration rate than the separation rate has been explained in Section 3.2.2.

### 3.4. Analysis of Concentration Performance

**Figure 7:**
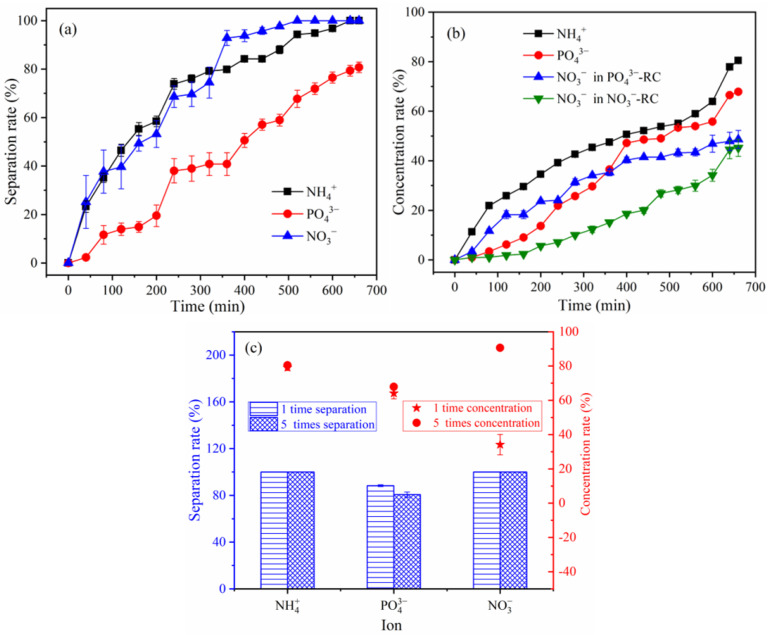
(**a**) separation and (**b**) concentration of NH_4_^+^, PO_4_^3−^ and NO_3_^−^ with the volume ratio of V_WWC_/V_AWC_, V_WWC_/V_PC_, and V_WWC_/V_NAC_ was 5:1 and (**c**) the comparison with volume ratio of V_WWC_/V_AWC_, V_WWC_/V_PC_, and V_WWC_/V_NAC_ was 1:1.

To validate the performance of the BMED in concentrating for NH_4_^+^, PO_4_^3−^, and NO_3_^−^, an experiment was performed with a volume ratio of V_WWC_/V_AWC_, V_WWC_/V_PC_, and V_WWC_/V_NAC_ (at a ratio of 5:1) (Figure 7), where V_WWC_, V_AWC_, V_PC_, and V_NAC_ represent the solution volume of WWC, AWC, PC and NAC, respectively.

As illustrated in Figure 7a,b, the separation and concentration rates of NH_4_^+^, PO_4_^3−^, and NO_3_^−^ increased with time. Both the NH_4_^+^ and NO_3_^−^ separation rates reached 100% after 660 min. In contrast, an 80.7% separation rate of PO_4_^3−^ was achieved. The concentration rate and concentration of NH_4_^+^ in AWC were 80.5% and 805 mg/L, respectively. PO_4_^3−^ in PC was present at a rate and concentration of 67.9% and 339 mg/L, respectively. NO_3_^−^ concentration rates in the PC and NAC were 48.6% and 45.2%, respectively, making up 93.8% of the total concentration rate. From Figure 7c, it was observed that the concentration rate of NH_4_^+^, PO_4_^3−^, and NO_3_^−^ increased compared with that mentioned in Section 3.2.2, when the volume ratio of V_WWC_/V_AWC_, V_WWC_/V_PC_, and V_WWC_/V_NAC_ was 1:1. This is due to the fact that with the increase of the wastewater volume, the total amount of NH_4_^+^, PO_4_^3−^, and NO_3_^−^ increased and the fraction of ions adsorbed by the membranes decreased so that a larger proportion of ions could be concentrated. According to Figure 7c, the separation rate of PO_4_^3−^ (80.7%) decreased slightly and the volume ratio of V_WWC_/V_AWC_, V_WWC_/V_PC_, and V_WWC_/V_NAC_ was 1:1 (i.e., 88.3%). This is primarily due to the fact that as time passed, the concentration of PO_4_^3−^ in the PC increased gradually, and the back-diffusion of PO_4_^3−^ from the PC to the WWC was reinforced, both of which would lower the separation rate of PO_4_^3−^. According to the aforementioned facts, the BMED system has a good performance from concentrating NH_4_^+^, PO_4_^3−^, and NO_3_^−^. NH_4_^+^ and PO_4_^3−^ concentrations could be increased to 805 mg/L and 339 mg/L, respectively.

### 3.5. Kinetics and Migration Rate Analysis

Zero-order, first-order, and second-order kinetic models were used to investigate the separation process of NH_4_^+^, PO_4_^3−^, and NO_3_^−^ removal in order to understand the kinetic process under various current densities (Table 1). The separation of NH_4_^+^, PO_4_^3−^, and NO_3_^−^ is more consistent with the zero-order kinetic process, that is, the separation rate of the three ions is independent of the initial concentration, because the *R*^2^ values corresponding to the zero-order kinetic equations under different current densities were higher than those of the first- and second-order kinetic models. The time required to achieve a certain separation rate is proportional to the initial concentration. Moreover, the ion migration rate is directly proportional to the current density, and the coefficient of kinetics (*k*_0_) corresponding to NH_4_^+^, PO_4_^3−^, and NO_3_^−^ increased with the current density. This shows that current density has a significant impact on the separation of the three ions. (*k*_0_) NH_4_^+^, PO_4_^3−^, and NO_3_^−^. When the current density increased from 0.5 to 4.0 mA/cm^2^, the *k*_0_ of NH_4_^+^ increased from 1.159 to 4.698 mg/L/min, while the *k*_0_ of PO_4_^3−^ and NO_3_^−^ increased from 0.407 to 1.735 mg/L/min and 0.185 to 0.457 mg/L/min, respectively.

**Figure 8:**
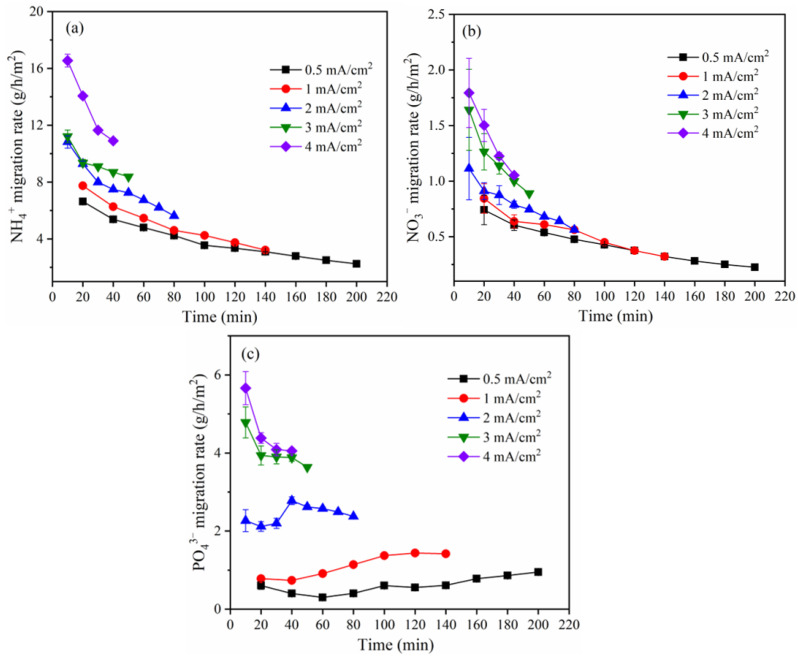
Migration rate of (**a**) NH_4_^+^, (**b**) PO_4_^3−^, and (**c**) NO_3_^−^ under different current densities.

The migration rate of NH_4_^+^ and NO_3_^−^ steadily decreased with time (Figure 8). The cause is that the ions had a high migration rate at the beginning due to the high ion concentration at the corresponding time. As more time passed, the ion concentration dropped, which caused the ion migratory rate to drop over time. Additionally, the ion migration rate was accelerated with the current density, mostly due to the fact that the electric field force on ions increased with current density. When the current density is 0.5, 1.0, 2.0, 3.0, and 4.0 mA/cm^2^, the average migration rate of NH_4_^+^ and NO_3_^−^ are 3.86, 5.04, 8.04, 9.35, 13.29 g/h/m^2^ and 0.42, 0.54, 0.79, 1.19, 1.39 g/h/m^2^, respectively. According to Figure 8c, the change in PO_4_^3−^ migration rate is complex, between current densities of 0.5 and 1.0 mA/cm^2^, the PO_4_^3−^ migration rate first decreased, then increased, while when the current density is ≥2.0 mA/cm^2^, the migration rate of PO_4_^3−^ first decreased and then increased, and finally decreased. Theoretically, the migration rate decreases with time, just as that of NH_4_^+^ and NO_3_^−^, but there was a migration competition between PO_4_^3−^ and NO_3_^−^, and since the NO_3_^−^ radius is much smaller than that of PO_4_^3−^, it was the dominant ion migrating to the anode initially, which might reduce PO_4_^3−^ migration rate. The migration competition between NO_3_^−^ with PO_4_^3−^ diminished over time as NO_3_^−^ concentration declined, which might increase PO_4_^3−^ migration. As PO_4_^3−^ concentration decreases, its migration rate would also decrease. The fluctuation in PO_4_^3−^ migration rate as shown in Figure 8c was achieved under the impact of the aforementioned parameters. When the current densities are 0.5, 1.0, 2.0, 3.0, and 4.0 mA/cm^2^, respectively, the average migration rate of PO_4_^3−^ were 0.61, 1.11, 2.43, 4.03, and 4.55 g/h/m^2^, respectively. A high current density can be used in the subsequent field engineering application process if a quick migration rate and short duration are required, but the energy consumption could be significant.

## 4. Summary

The primary findings of this study, which used selective BMED to separate and concentrate nitrogen and phosphorus in simulated wastewater, are as follows.

(1) The separation rate and migration rate of NH_4_^+^, PO_4_^3−^, and NO_3_^−^ increased with current density. When the current density was 1.0 mA/cm^2^, the separation rates of NH_4_^+^, PO_4_^3−^, and NO_3_^−^ after 140 min were 100%, 88.3%, and 100%, respectively. Their concentration rates were much lower than the separation rates due to membrane adsorption. The recoveries of them were 79.1%, 64.1%, and 34.2%, respectively.

(2) Organic matter had no effect on ion separation and concentration. The same separation rate and concentration rate of NH_4_^+^, PO_4_^3−^, and NO_3_^−^ were achieved under the condition of different organic matter content.

(3) MVA had a good performance for separating NO_3_^−^ and PO_4_^3−^, and PO_4_^3−^ was not detected in nitric acid compartment.

(4) The migration process of NH_4_^+^, PO_4_^3−^, and NO_3_^−^ followed a zero-order kinetic process, and their migration rate was independent of the initial concentration.

(5) The findings of the multifold concentration show that the BMED was able to separate and concentrate the NH_4_^+^, PO_4_^3−^, and NO_3_^−^. Thus, the BMED system can be regarded as an effective method to separate and concentrate the NH_4_^+^, PO_4_^3−^, and NO_3_^−^ from wastewater.

## Figures and Tables

**Table 1:** Migration kinetic analysis of NH_4_^+^, PO_4_^3−^, and NO_3_^−^.

Type of Ions	Type of Dynamics	Coefficient of Kinetics k and R^2^	Current Density (mA/cm^2^)				
			0.5	1.0	2.0	3.0	4.0
NH_4_^+^	Zero-order	k_0_ (mg/L/min)	1.159	1.804	2.628	3.666	4.698
		R^2^	0.913	0.944	0.966	0.994	0.969
	First-order e	k_1_ (min^−1^)	0.026	0.026	0.043	0.049	0.080
		R^2^	0.885	0.951	0.914	0.904	0.876
	Second-order	k_2_ (L/mg/min)	0.002	0.000	0.001	0.001	0.003
		R^2^	0.438	0.676	0.588	0.644	0.592
PO_4_^3−^	Zero-order	k_0_ (mg/L/min)	0.407	0.676	1.114	1.612	1.735
		R^2^	0.918	0.980	0.989	0.994	0.989
	First-order	k_1_ (min^−1^)	0.007	0.014	0.023	0.032	0.030
		R^2^	0.773	0.881	0.963	0.968	0.973
	Second-order	k_2_ (L/mg/min)	0.000	0.000	0.000	0.000	0.000
		R^2^	0.567	0.680	0.830	0.851	0.885
NO_3_^−^	Zero-order	k_0_ (mg/L/min)	0.185	0.263	0.277	0.382	0.457
		R^2^	0.958	0.980	0.970	0.951	0.939
	First-order	k_1_ (min^−1^)	0.028	0.027	0.063	0.078	0.066
		R^2^	0.952	0.965	0.759	0.875	0.977
	Second-order	k_2_ (L/mg/min)	0.007	0.003	0.107	0.057	0.016
		R^2^	0.681	0.844	0.364	0.514	0.760

## Data Availability

The data presented in this study are available on request from the corresponding author.

## Supplementary Materials

The following are available online at https://www.mdpi.com/article/10.3390/membranes12111116/s1, Figure S1: Changes of solution conductivity in (a) wastewater compartment, (b) ammonia compartment, (c) phosphate compartment and (d) nitric acid compartment under different current density, Table S1: Characteristics of membranes.

## Abbreviations

CC: cathode compartment; AWC: ammonia compartment; WWC: wastewater compartment; PC: phosphate compartment; NAC: nitric acid compartment; AC: anode compartment; SBMED: selective BMED; MVA: monovalent selective anion exchange membranes; SED: selective selectrodialysis; BPM: bipolar membrane; CEM: cation exchange membrane; AEM: anion exchange membrane.
