# Supplementary material for: Separation and Concentration of Nitrogen and Phosphorus in a Bipolar Membrane Electrodialysis System
Source: Membranes (Basel). 2022 Nov 8;12(11):1116. doi: 10.3390/membranes12111116 (PMC9695792; doi:10.3390/membranes12111116)
Supplement: Supplementary file 1 [file membranes-12-01116-s001.zip › membranes-1990201-supplementary.pdf]

membranes

MDPI

Supplementary Materials

Separation and Concentration of Nitrogen and Phosphorus in a Bipolar Membrane Electrodialysis System

S1 Details of membranes

Table S1. Characteristics of membranes.

Parameters	CEM	AEM	BPM
Thickness (μm)	50.8	420	170–260
Conductivity (S/cm)	0.083	0.083	-
Exchange capacity (mol/kg)	0.95–1.01	2.2	-
Moisture content (%)	5 ± 3.0	42	-
Rupture strength (MPa)	-	0.6	0.4–0.7
Selective transmission coefficient (%)	-	90	-
Water decomposition voltage (V)	-	-	0.9–1.7
Water decomposition efficiency (%)	-	-	>98

S2 Changes of pH in different compartment

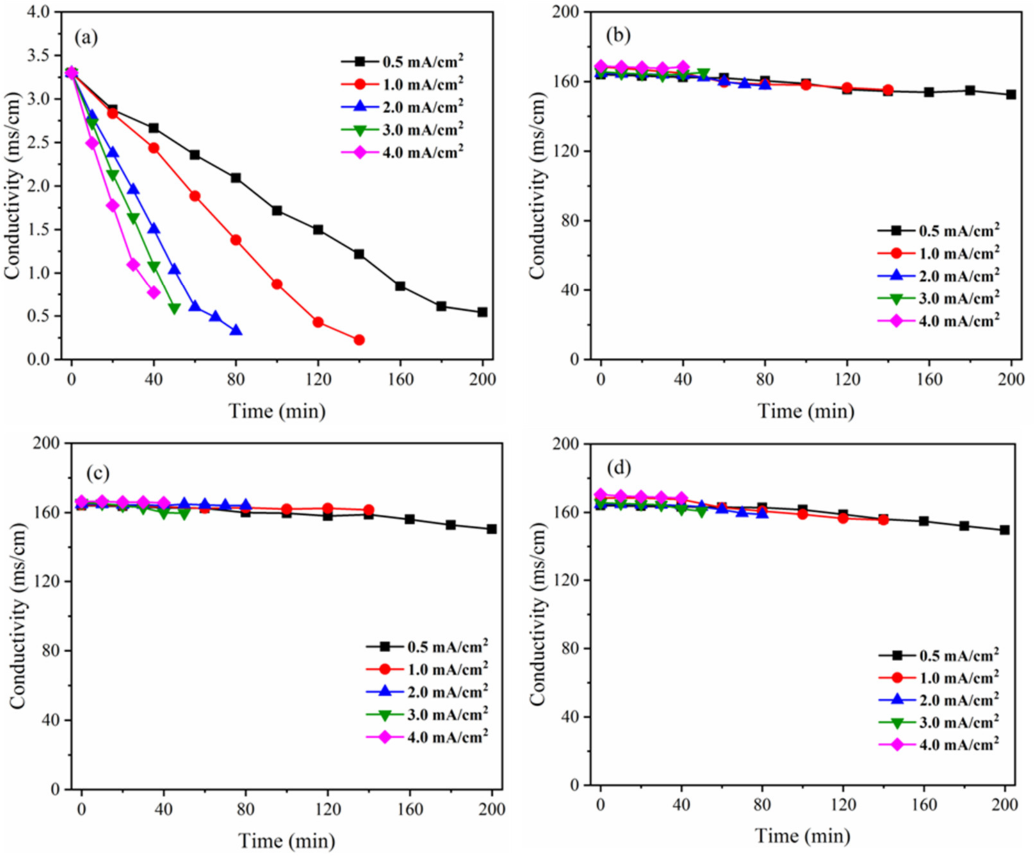

Figure S1. Changes of solution conductivity in (**a**) wastewater compartment, (**b**) ammonia compartment, (**c**) phosphate compartment and (**d**) nitric acid compartment under different current density.
